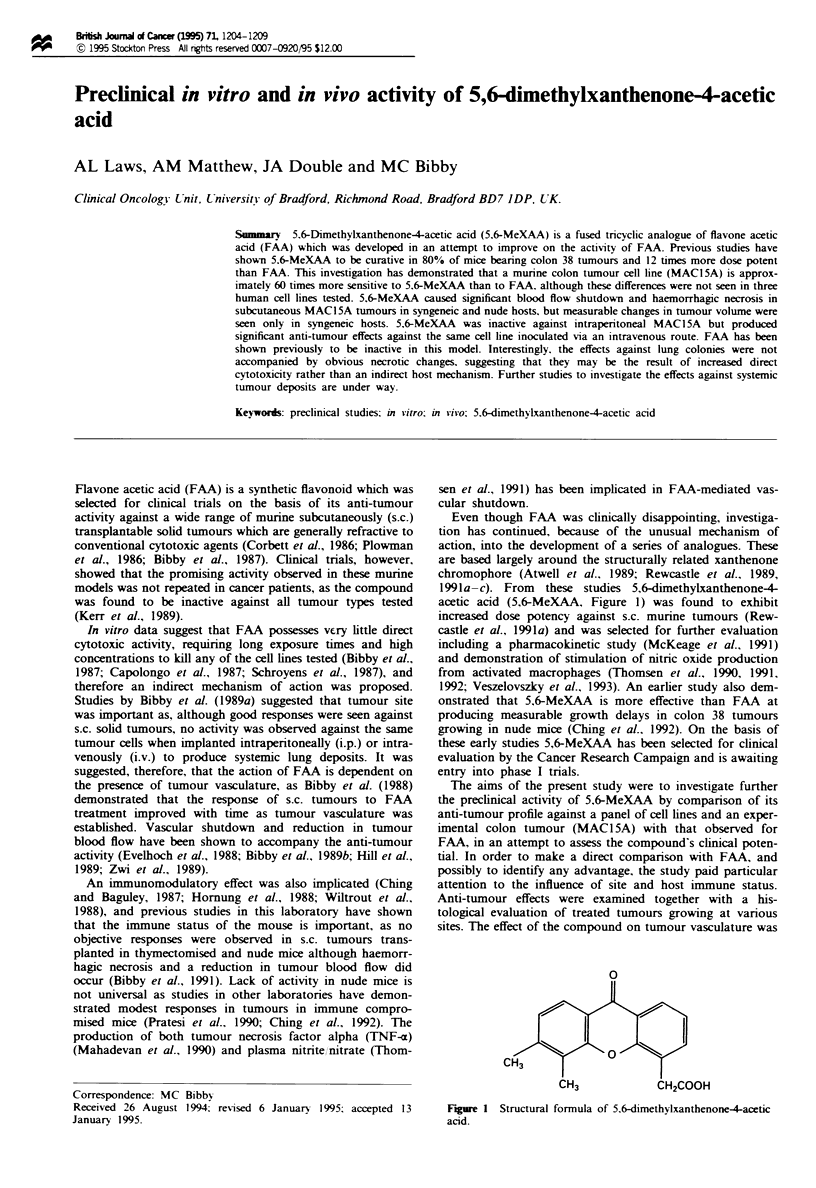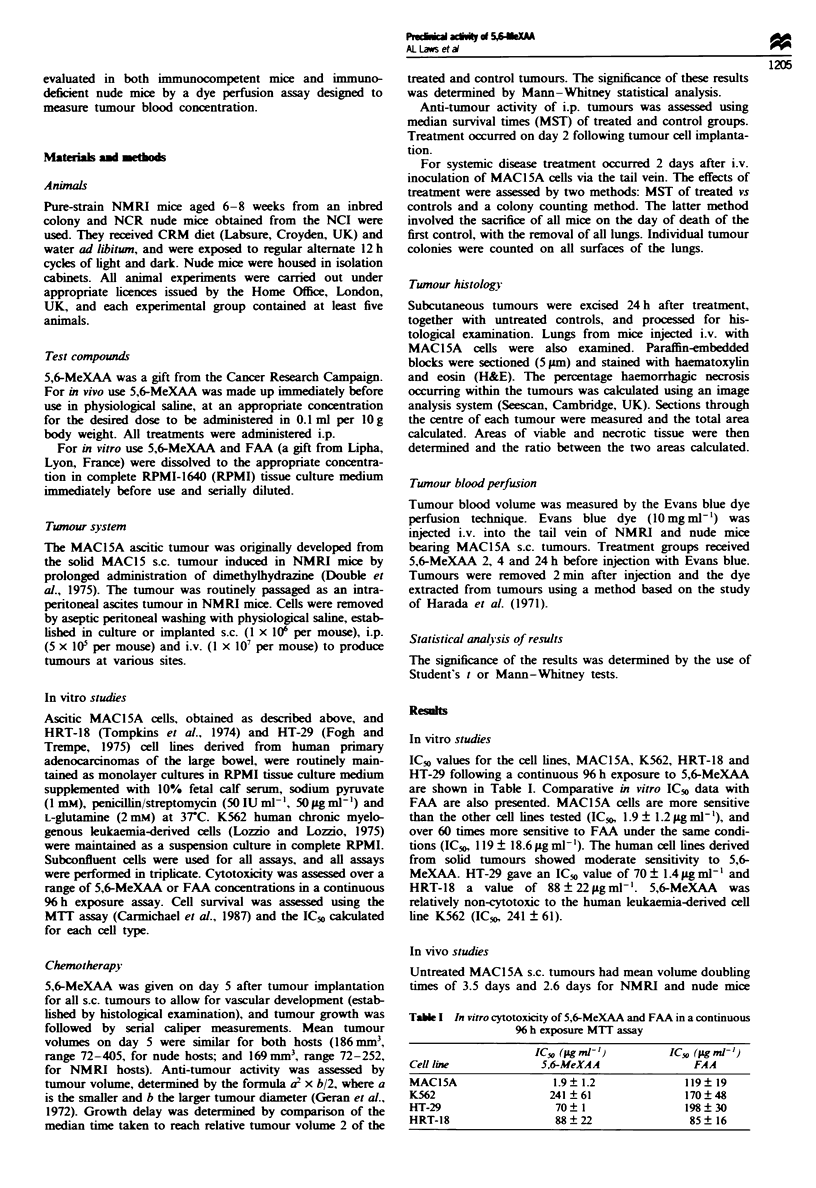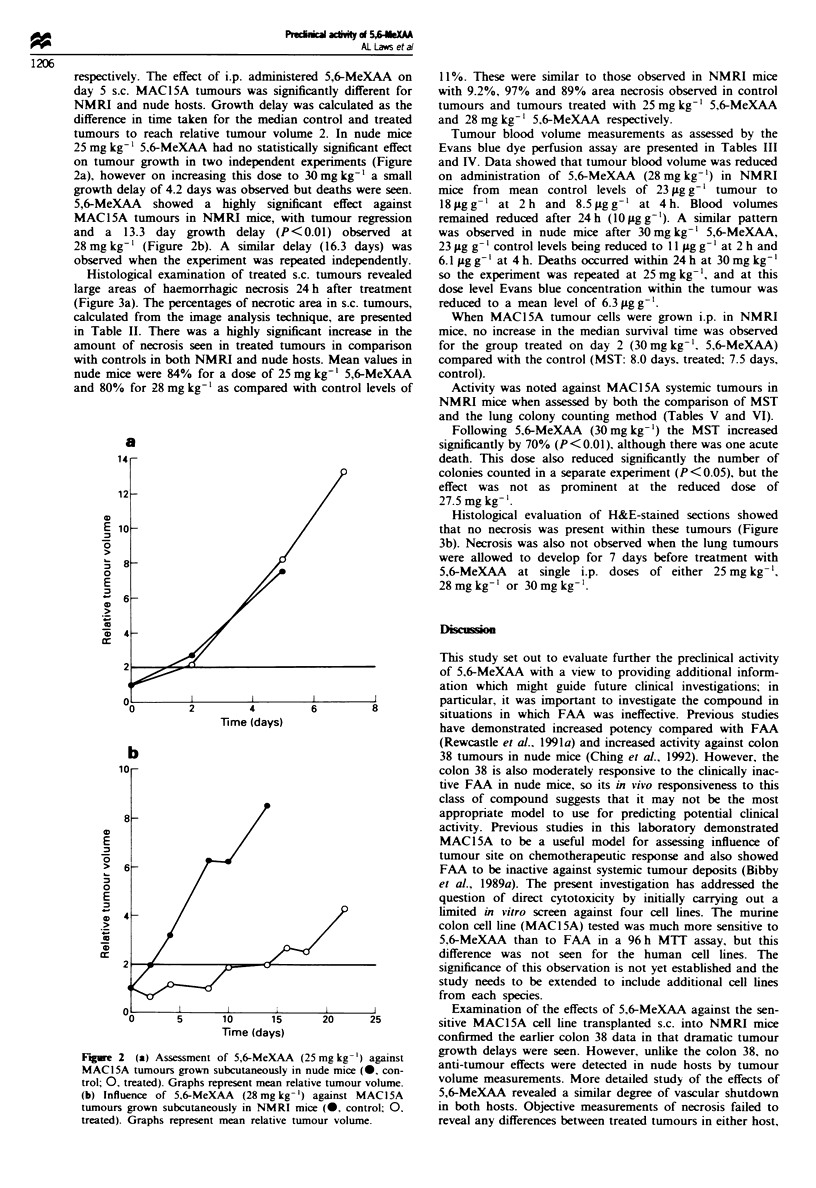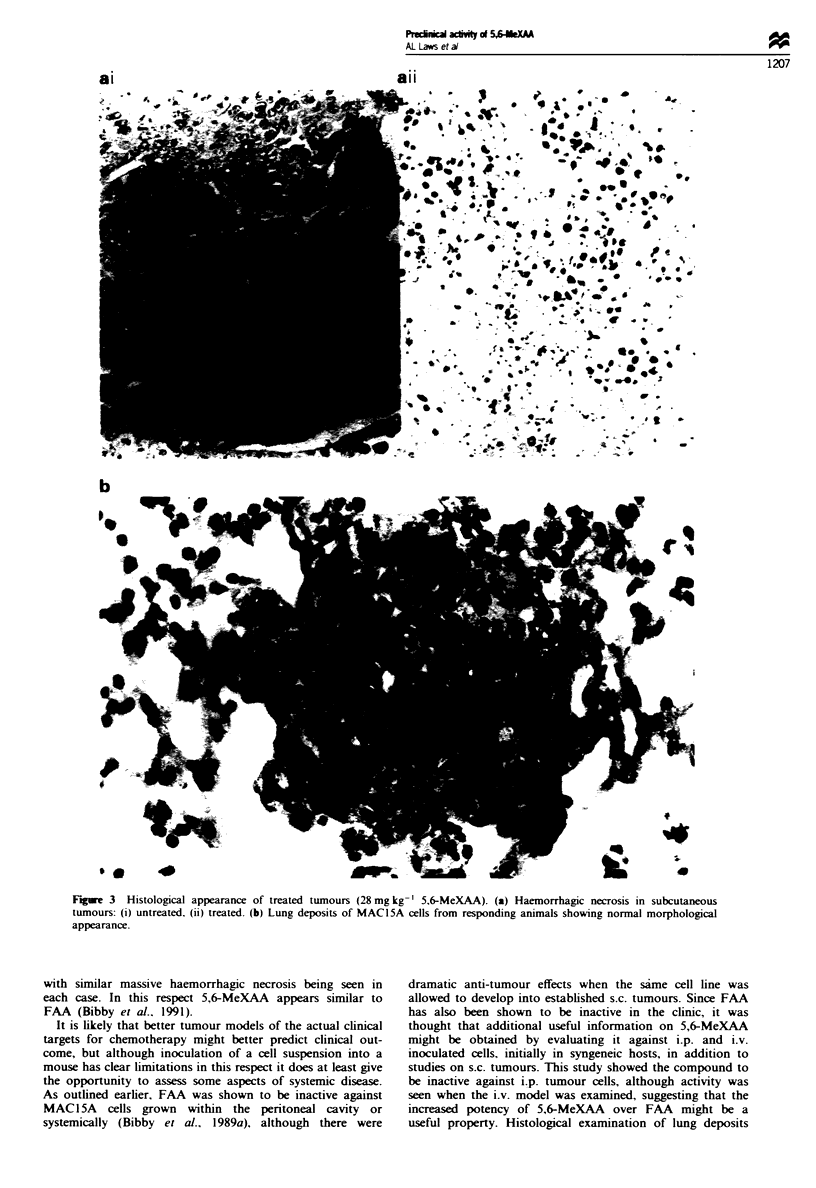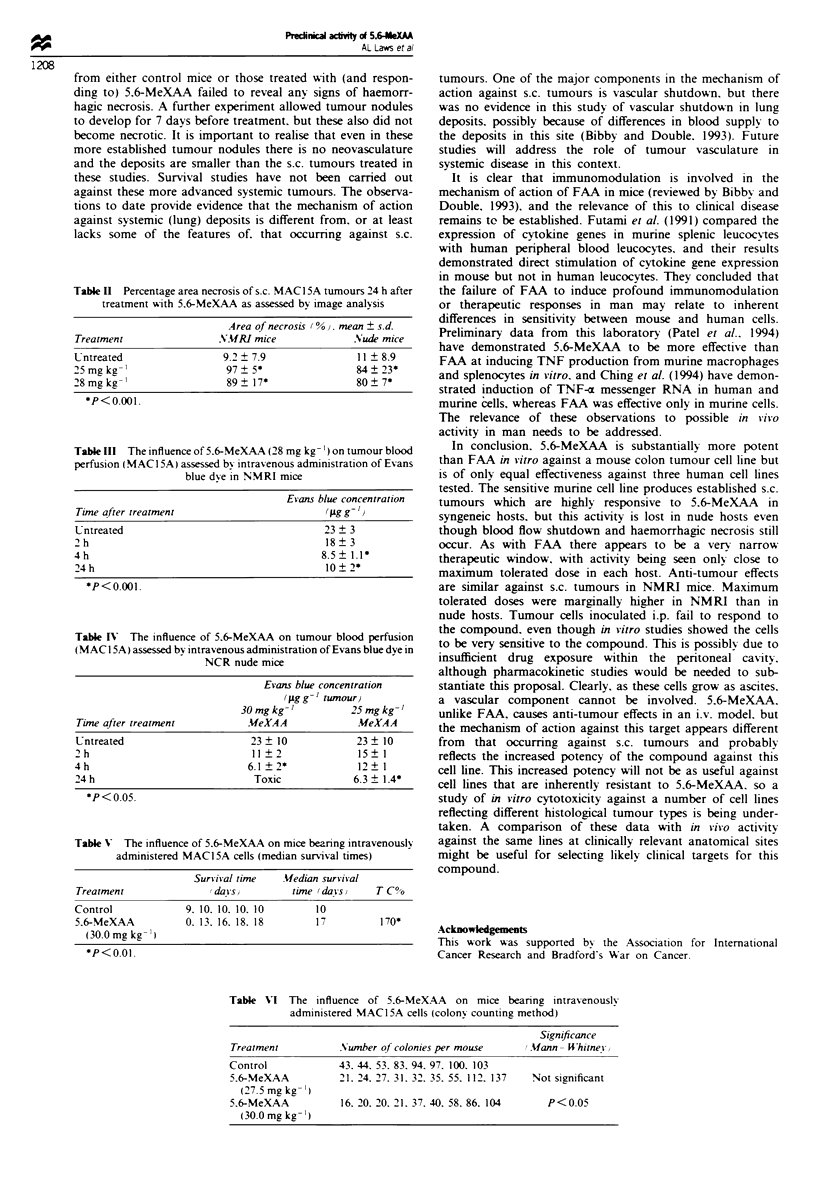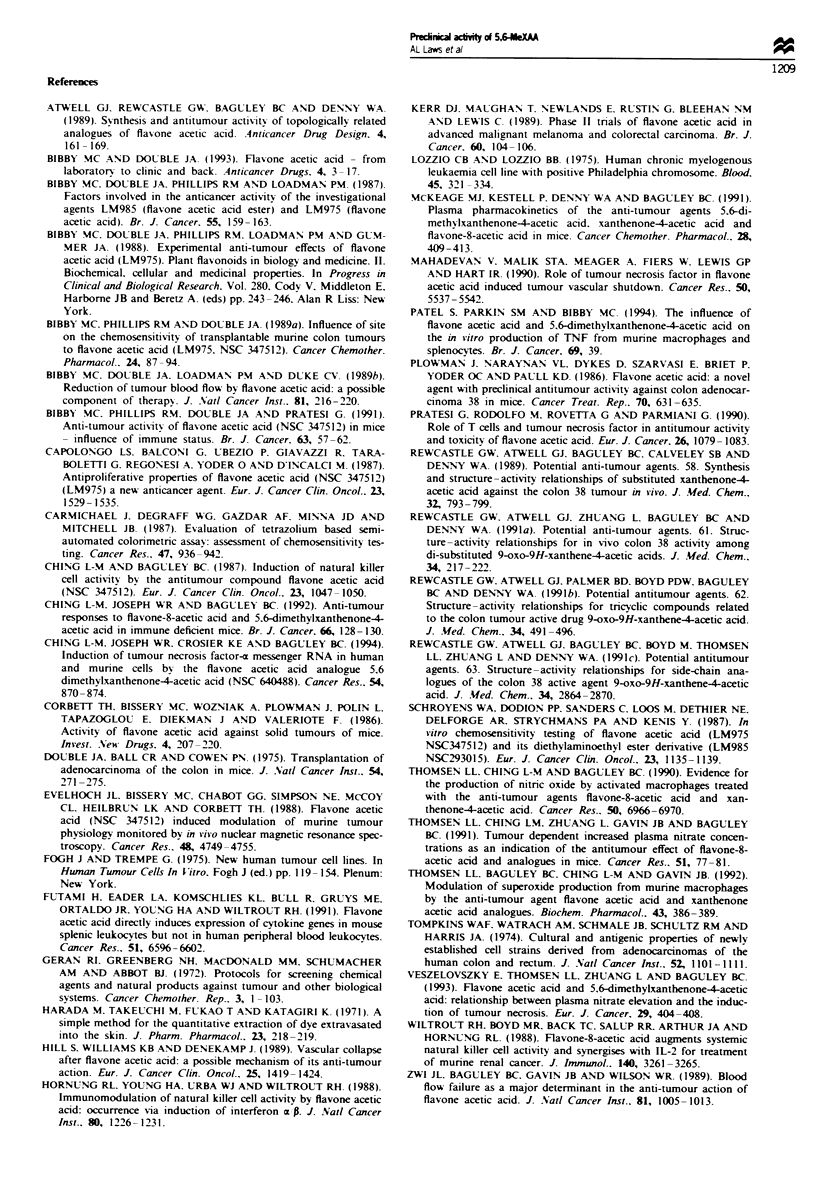# Preclinical in vitro and in vivo activity of 5,6-dimethylxanthenone-4-acetic acid.

**DOI:** 10.1038/bjc.1995.234

**Published:** 1995-06

**Authors:** A. L. Laws, A. M. Matthew, J. A. Double, M. C. Bibby

**Affiliations:** Clinical Oncology Unit, University of Bradford, UK.

## Abstract

**Images:**


British Journal of Cancer (1995) 71, 1204–1209

© 1995 Stockton Press All rights reserved 0007–0920/95 $12.00

### Preclinical *in vitro* and *in vivo* activity of 5,6-dimethylxanthenone-4-acetic acid

AL Laws, AM Matthew, JA Double and MC Bibby

*Clinical Oncology Unit, University of Bradford, Richmond Road, Bradford BD7 1DP, UK.*

**Summary** 5,6-Dimethylxanthenone-4-acetic acid (5,6-MeXAA) is a fused tricyclic analogue of flavone acetic acid (FAA) which was developed in an attempt to improve on the activity of FAA. Previous studies have shown 5,6-MeXAA to be curative in 80% of mice bearing colon 38 tumours and 12 times more dose potent than FAA. This investigation has demonstrated that a murine colon tumour cell line (MAC15A) is approximately 60 times more sensitive to 5,6-MeXAA than to FAA, although these differences were not seen in three human cell lines tested. 5,6-MeXAA caused significant blood flow shutdown and haemorrhagic necrosis in subcutaneous MAC15A tumours in syngeneic and nude hosts, but measurable changes in tumour volume were seen only in syngeneic hosts. 5,6-MeXAA was inactive against intraperitoneal MAC15A but produced significant anti-tumour effects against the same cell line inoculated via an intravenous route. FAA has been shown previously to be inactive in this model. Interestingly, the effects against lung colonies were not accompanied by obvious necrotic changes, suggesting that they may be the result of increased direct cytotoxicity rather than an indirect host mechanism. Further studies to investigate the effects against systemic tumour deposits are under way.

**Keywords**: preclinical studies; *in vitro*; *in vivo*; 5,6-dimethylxanthenone-4-acetic acid

Flavone acetic acid (FAA) is a synthetic flavonoid which was selected for clinical trials on the basis of its anti-tumour activity against a wide range of murine subcutaneously (s.c.) transplantable solid tumours which are generally refractive to conventional cytotoxic agents (Corbett *et al*., 1986; Plowman *et al*., 1986; Bibby *et al*., 1987). Clinical trials, however, showed that the promising activity observed in these murine models was not repeated in cancer patients, as the compound was found to be inactive against all tumour types tested (Kerr *et al*., 1989).

*In vitro* data suggest that FAA possesses very little direct cytotoxic activity, requiring long exposure times and high concentrations to kill any of the cell lines tested (Bibby *et al*., 1987; Capolongo *et al*., 1987; Schroyens *et al*., 1987), and therefore an indirect mechanism of action was proposed. Studies by Bibby *et al*. (1989*a*) suggested that tumour site was important as, although good responses were seen against s.c. solid tumours, no activity was observed against the same tumour cells when implanted intraperitoneally (i.p.) or intravenously (i.v.) to produce systemic lung deposits. It was suggested, therefore, that the action of FAA is dependent on the presence of tumour vasculature, as Bibby *et al*. (1988) demonstrated that the response of s.c. tumours to FAA treatment improved with time as tumour vasculature was established. Vascular shutdown and reduction in tumour blood flow have been shown to accompany the anti-tumour activity (Evelhoch *et al*., 1988; Bibby *et al*., 1989*b*; Hill *et al*., 1989; Zwi *et al*., 1989).

An immunomodulatory effect was also implicated (Ching and Baguley, 1987; Hornung *et al*., 1988; Wiltrout *et al*., 1988), and previous studies in this laboratory have shown that the immune status of the mouse is important, as no objective responses were observed in s.c. tumours transplanted in thymectomised and nude mice although haemorrhagic necrosis and a reduction in tumour blood flow did occur (Bibby *et al*., 1991). Lack of activity in nude mice is not universal as studies in other laboratories have demonstrated modest responses in tumours in immune compromised mice (Pratesi *et al*., 1990; Ching *et al*., 1992). The production of both tumour necrosis factor alpha (TNF-α) (Mahadevan *et al*., 1990) and plasma nitrite/nitrate (Thomsen *et al*., 1991) has been implicated in FAA-mediated vascular shutdown.

Even though FAA was clinically disappointing, investigation has continued, because of the unusual mechanism of action, into the development of a series of analogues. These are based largely around the structurally related xanthenone chromophore (Atwell *et al*., 1989; Rewcastle *et al*., 1989, 1991*a–c*). From these studies 5,6-dimethylxanthenone-4-acetic acid (5,6-MeXAA, Figure 1) was found to exhibit increased dose potency against s.c. murine tumours (Rewcastle *et al*., 1991*a*) and was selected for further evaluation including a pharmacokinetic study (McKeage *et al*., 1991) and demonstration of stimulation of nitric oxide production from activated macrophages (Thomsen *et al*., 1990, 1991, 1992; Veszelovszky *et al*., 1993). An earlier study also demonstrated that 5,6-MeXAA is more effective than FAA at producing measurable growth delays in colon 38 tumours growing in nude mice (Ching *et al*., 1992). On the basis of these early studies 5,6-MeXAA has been selected for clinical evaluation by the Cancer Research Campaign and is awaiting entry into phase I trials.

The aims of the present study were to investigate further the preclinical activity of 5,6-MeXAA by comparison of its anti-tumour profile against a panel of cell lines and an experimental colon tumour (MAC15A) with that observed for FAA, in an attempt to assess the compound's clinical potential. In order to make a direct comparison with FAA, and possibly to identify any advantage, the study paid particular attention to the influence of site and host immune status. Anti-tumour effects were examined together with a histological evaluation of treated tumours growing at various sites. The effect of the compound on tumour vasculature was

**Figure 1** Structural formula of 5,6-dimethylxanthenone-4-acetic acid.

Correspondence: MC Bibby
Received 26 August 1994; revised 6 January 1995; accepted 13 January 1995.

evaluated in both immunocompetent mice and immunodeficient nude mice by a dye perfusion assay designed to measure tumour blood concentration.

### Materials and methods

### Animals

Pure-strain NMRI mice aged 6–8 weeks from an inbred colony and NCR nude mice obtained from the NCI were used. They received CRM diet (Labsure, Croyden, UK) and water *ad libitum*, and were exposed to regular alternate 12 h cycles of light and dark. Nude mice were housed in isolation cabinets. All animal experiments were carried out under appropriate licences issued by the Home Office, London, UK, and each experimental group contained at least five animals.

### Test compounds

5,6-MeXAA was a gift from the Cancer Research Campaign. For *in vivo* use 5,6-MeXAA was made up immediately before use in physiological saline, at an appropriate concentration for the desired dose to be administered in 0.1 ml per 10 g body weight. All treatments were administered i.p.

For *in vitro* use 5,6-MeXAA and FAA (a gift from Lipha, Lyon, France) were dissolved to the appropriate concentration in complete RPMI-1640 (RPMI) tissue culture medium immediately before use and serially diluted.

### Tumour system

The MAC15A ascitic tumour was originally developed from the solid MAC15 s.c. tumour induced in NMRI mice by prolonged administration of dimethylhydrazine (Double *et al.*, 1975). The tumour was routinely passaged as an intraperitoneal ascites tumour in NMRI mice. Cells were removed by aseptic peritoneal washing with physiological saline, established in culture or implanted s.c. ($1 \times 10^6$ per mouse), i.p. ($5 \times 10^5$ per mouse) and i.v. ($1 \times 10^7$ per mouse) to produce tumours at various sites.

### In vitro *studies*

Ascitic MAC15A cells, obtained as described above, and HRT-18 (Tompkins *et al.*, 1974) and HT-29 (Fogh and Trempe, 1975) cell lines derived from human primary adenocarcinomas of the large bowel, were routinely maintained as monolayer cultures in RPMI tissue culture medium supplemented with 10% fetal calf serum, sodium pyruvate (1 mM), penicillin/streptomycin (50 IU $ml^{-1}$, 50 $\mu g\ ml^{-1}$) and L-glutamine (2 mM) at 37°C. K562 human chronic myelogenous leukaemia-derived cells (Lozzio and Lozzio, 1975) were maintained as a suspension culture in complete RPMI. Subconfluent cells were used for all assays, and all assays were performed in triplicate. Cytotoxicity was assessed over a range of 5,6-MeXAA or FAA concentrations in a continuous 96 h exposure assay. Cell survival was assessed using the MTT assay (Carmichael *et al.*, 1987) and the $IC_{50}$ calculated for each cell type.

### Chemotherapy

5,6-MeXAA was given on day 5 after tumour implantation for all s.c. tumours to allow for vascular development (established by histological examination), and tumour growth was followed by serial caliper measurements. Mean tumour volumes on day 5 were similar for both hosts (186 $mm^3$, range 72–405, for nude hosts; and 169 $mm^3$, range 72–252, for NMRI hosts). Anti-tumour activity was assessed by tumour volume, determined by the formula $a^2 \times b/2$, where $a$ is the smaller and $b$ the larger tumour diameter (Geran *et al.*, 1972). Growth delay was determined by comparison of the median time taken to reach relative tumour volume 2 of the treated and control tumours. The significance of these results was determined by Mann–Whitney statistical analysis.

Anti-tumour activity of i.p. tumours was assessed using median survival times (MST) of treated and control groups. Treatment occurred on day 2 following tumour cell implantation.

For systemic disease treatment occurred 2 days after i.v. inoculation of MAC15A cells via the tail vein. The effects of treatment were assessed by two methods: MST of treated *vs* controls and a colony counting method. The latter method involved the sacrifice of all mice on the day of death of the first control, with the removal of all lungs. Individual tumour colonies were counted on all surfaces of the lungs.

### Tumour histology

Subcutaneous tumours were excised 24 h after treatment, together with untreated controls, and processed for histological examination. Lungs from mice injected i.v. with MAC15A cells were also examined. Paraffin-embedded blocks were sectioned (5 μm) and stained with haematoxylin and eosin (H&E). The percentage haemorrhagic necrosis occurring within the tumours was calculated using an image analysis system (Seescan, Cambridge, UK). Sections through the centre of each tumour were measured and the total area calculated. Areas of viable and necrotic tissue were then determined and the ratio between the two areas calculated.

### Tumour blood perfusion

Tumour blood volume was measured by the Evans blue dye perfusion technique. Evans blue dye (10 mg $ml^{-1}$) was injected i.v. into the tail vein of NMRI and nude mice bearing MAC15A s.c. tumours. Treatment groups received 5,6-MeXAA 2, 4 and 24 h before injection with Evans blue. Tumours were removed 2 min after injection and the dye extracted from tumours using a method based on the study of Harada *et al.* (1971).

### Statistical analysis of results

The significance of the results was determined by the use of Student's $t$ or Mann–Whitney tests.

### Results

### In vitro *studies*

$IC_{50}$ values for the cell lines, MAC15A, K562, HRT-18 and HT-29 following a continuous 96 h exposure to 5,6-MeXAA are shown in Table I. Comparative *in vitro* $IC_{50}$ data with FAA are also presented. MAC15A cells are more sensitive than the other cell lines tested ($IC_{50}$, 1.9 ± 1.2 $\mu g\ ml^{-1}$), and over 60 times more sensitive to FAA under the same conditions ($IC_{50}$, 119 ± 18.6 $\mu g\ ml^{-1}$). The human cell lines derived from solid tumours showed moderate sensitivity to 5,6-MeXAA. HT-29 gave an $IC_{50}$ value of 70 ± 1.4 $\mu g\ ml^{-1}$ and HRT-18 a value of 88 ± 22 $\mu g\ ml^{-1}$. 5,6-MeXAA was relatively non-cytotoxic to the human leukaemia-derived cell line K562 ($IC_{50}$, 241 ± 61).

### In vivo *studies*

Untreated MAC15A s.c. tumours had mean volume doubling times of 3.5 days and 2.6 days for NMRI and nude mice

**Table I** *In vitro* cytotoxicity of 5,6-MeXAA and FAA in a continuous 96 h exposure MTT assay

| *Cell line* | *$IC_{50}$ ($\mu g\ ml^{-1}$) 5,6-MeXAA* | *$IC_{50}$ ($\mu g\ ml^{-1}$) FAA* |
|---|---|---|
| MAC15A | 1.9 ± 1.2 | 119 ± 19 |
| K562 | 241 ± 61 | 170 ± 48 |
| HT-29 | 70 ± 1 | 198 ± 30 |
| HRT-18 | 88 ± 22 | 85 ± 16 |

respectively. The effect of i.p. administered 5,6-MeXAA on day 5 s.c. MAC15A tumours was significantly different for NMRI and nude hosts. Growth delay was calculated as the difference in time taken for the median control and treated tumours to reach relative tumour volume 2. In nude mice 25 mg $kg^{-1}$ 5,6-MeXAA had no statistically significant effect on tumour growth in two independent experiments (Figure 2a), however on increasing this dose to 30 mg $kg^{-1}$ a small growth delay of 4.2 days was observed but deaths were seen. 5,6-MeXAA showed a highly significant effect against MAC15A tumours in NMRI mice, with tumour regression and a 13.3 day growth delay ($P<0.01$) observed at 28 mg $kg^{-1}$ (Figure 2b). A similar delay (16.3 days) was observed when the experiment was repeated independently.

Histological examination of treated s.c. tumours revealed large areas of haemorrhagic necrosis 24 h after treatment (Figure 3a). The percentages of necrotic area in s.c. tumours, calculated from the image analysis technique, are presented in Table II. There was a highly significant increase in the amount of necrosis seen in treated tumours in comparison with controls in both NMRI and nude hosts. Mean values in nude mice were 84% for a dose of 25 mg $kg^{-1}$ 5,6-MeXAA and 80% for 28 mg $kg^{-1}$ as compared with control levels of

**Figure 2** (**a**) Assessment of 5,6-MeXAA (25 mg $kg^{-1}$) against MAC15A tumours grown subcutaneously in nude mice (●, control; O, treated). Graphs represent mean relative tumour volume. (**b**) Influence of 5,6-MeXAA (28 mg $kg^{-1}$) against MAC15A tumours grown subcutaneously in NMRI mice (●, control; O, treated). Graphs represent mean relative tumour volume.

11%. These were similar to those observed in NMRI mice with 9.2%, 97% and 89% area necrosis observed in control tumours and tumours treated with 25 mg $kg^{-1}$ 5,6-MeXAA and 28 mg $kg^{-1}$ 5,6-MeXAA respectively.

Tumour blood volume measurements as assessed by the Evans blue dye perfusion assay are presented in Tables III and IV. Data showed that tumour blood volume was reduced on administration of 5,6-MeXAA (28 mg $kg^{-1}$) in NMRI mice from mean control levels of 23 $\mu g\ g^{-1}$ tumour to 18 $\mu g\ g^{-1}$ at 2 h and 8.5 $\mu g\ g^{-1}$ at 4 h. Blood volumes remained reduced after 24 h (10 $\mu g\ g^{-1}$). A similar pattern was observed in nude mice after 30 mg $kg^{-1}$ 5,6-MeXAA, 23 $\mu g\ g^{-1}$ control levels being reduced to 11 $\mu g\ g^{-1}$ at 2 h and 6.1 $\mu g\ g^{-1}$ at 4 h. Deaths occurred within 24 h at 30 mg $kg^{-1}$ so the experiment was repeated at 25 mg $kg^{-1}$, and at this dose level Evans blue concentration within the tumour was reduced to a mean level of 6.3 $\mu g\ g^{-1}$.

When MAC15A tumour cells were grown i.p. in NMRI mice, no increase in the median survival time was observed for the group treated on day 2 (30 mg $kg^{-1}$, 5,6-MeXAA) compared with the control (MST: 8.0 days, treated; 7.5 days, control).

Activity was noted against MAC15A systemic tumours in NMRI mice when assessed by both the comparison of MST and the lung colony counting method (Tables V and VI).

Following 5,6-MeXAA (30 mg $kg^{-1}$) the MST increased significantly by 70% ($P<0.01$), although there was one acute death. This dose also reduced significantly the number of colonies counted in a separate experiment ($P<0.05$), but the effect was not as prominent at the reduced dose of 27.5 mg $kg^{-1}$.

Histological evaluation of H&E-stained sections showed that no necrosis was present within these tumours (Figure 3b). Necrosis was also not observed when the lung tumours were allowed to develop for 7 days before treatment with 5,6-MeXAA at single i.p. doses of either 25 mg $kg^{-1}$, 28 mg $kg^{-1}$ or 30 mg $kg^{-1}$.

### Discussion

This study set out to evaluate further the preclinical activity of 5,6-MeXAA with a view to providing additional information which might guide future clinical investigations; in particular, it was important to investigate the compound in situations in which FAA was ineffective. Previous studies have demonstrated increased potency compared with FAA (Rewcastle *et al.*, 1991*a*) and increased activity against colon 38 tumours in nude mice (Ching *et al.*, 1992). However, the colon 38 is also moderately responsive to the clinically inactive FAA in nude mice, so its *in vivo* responsiveness to this class of compound suggests that it may not be the most appropriate model to use for predicting potential clinical activity. Previous studies in this laboratory demonstrated MAC15A to be a useful model for assessing influence of tumour site on chemotherapeutic response and also showed FAA to be inactive against systemic tumour deposits (Bibby *et al.*, 1989*a*). The present investigation has addressed the question of direct cytotoxicity by initially carrying out a limited *in vitro* screen against four cell lines. The murine colon cell line (MAC15A) tested was much more sensitive to 5,6-MeXAA than to FAA in a 96 h MTT assay, but this difference was not seen for the human cell lines. The significance of this observation is not yet established and the study needs to be extended to include additional cell lines from each species.

Examination of the effects of 5,6-MeXAA against the sensitive MAC15A cell line transplanted s.c. into NMRI mice confirmed the earlier colon 38 data in that dramatic tumour growth delays were seen. However, unlike the colon 38, no anti-tumour effects were detected in nude hosts by tumour volume measurements. More detailed study of the effects of 5,6-MeXAA revealed a similar degree of vascular shutdown in both hosts. Objective measurements of necrosis failed to reveal any differences between treated tumours in either host, ai aii b

**Figure 3** Histological appearance of treated tumours (28 mg $kg^{-1}$ 5,6-MeXAA). (**a**) Haemorrhagic necrosis in subcutaneous tumours: (i) untreated, (ii) treated. (**b**) Lung deposits of MAC15A cells from responding animals showing normal morphological appearance.

with similar massive haemorrhagic necrosis being seen in each case. In this respect 5,6-MeXAA appears similar to FAA (Bibby *et al.*, 1991).

It is likely that better tumour models of the actual clinical targets for chemotherapy might better predict clinical outcome, but although inoculation of a cell suspension into a mouse has clear limitations in this respect it does at least give the opportunity to assess some aspects of systemic disease. As outlined earlier, FAA was shown to be inactive against MAC15A cells grown within the peritoneal cavity or systemically (Bibby *et al.*, 1989*a*), although there were dramatic anti-tumour effects when the same cell line was allowed to develop into established s.c. tumours. Since FAA has also been shown to be inactive in the clinic, it was thought that additional useful information on 5,6-MeXAA might be obtained by evaluating it against i.p. and i.v. inoculated cells, initially in syngeneic hosts, in addition to studies on s.c. tumours. This study showed the compound to be inactive against i.p. tumour cells, although activity was seen when the i.v. model was examined, suggesting that the increased potency of 5,6-MeXAA over FAA might be a useful property. Histological examination of lung deposits from either control mice or those treated with (and responding to) 5,6-MeXAA failed to reveal any signs of haemorrhagic necrosis. A further experiment allowed tumour nodules to develop for 7 days before treatment, but these also did not become necrotic. It is important to realise that even in these more established tumour nodules there is no neovasculature and the deposits are smaller than the s.c. tumours treated in these studies. Survival studies have not been carried out against these more advanced systemic tumours. The observations to date provide evidence that the mechanism of action against systemic (lung) deposits is different from, or at least lacks some of the features of, that occurring against s.c. tumours. One of the major components in the mechanism of action against s.c. tumours is vascular shutdown, but there was no evidence in this study of vascular shutdown in lung deposits, possibly because of differences in blood supply to the deposits in this site (Bibby and Double, 1993). Future studies will address the role of tumour vasculature in systemic disease in this context.

**Table II** Percentage area necrosis of s.c. MAC15A tumours 24 h after treatment with 5,6-MeXAA as assessed by image analysis

| Treatment | Area of necrosis (%), mean ± s.d. NMRI mice | Nude mice |
|---|---|---|
| Untreated | 9.2 ± 7.9 | 11 ± 8.9 |
| 25 mg kg$^{-1}$ | 97 ± 5* | 84 ± 23* |
| 28 mg kg$^{-1}$ | 89 ± 17* | 80 ± 7* |

*$P<0.001$.

**Table III** The influence of 5,6-MeXAA (28 mg kg$^{-1}$) on tumour blood perfusion (MAC15A) assessed by intravenous administration of Evans blue dye in NMRI mice

| Time after treatment | Evans blue concentration (μg g$^{-1}$) |
|---|---|
| Untreated | 23 ± 3 |
| 2 h | 18 ± 3 |
| 4 h | 8.5 ± 1.1* |
| 24 h | 10 ± 2* |

*$P<0.001$.

**Table IV** The influence of 5,6-MeXAA on tumour blood perfusion (MAC15A) assessed by intravenous administration of Evans blue dye in NCR nude mice

| Time after treatment | Evans blue concentration (μg g$^{-1}$ tumour) 30 mg kg$^{-1}$ MeXAA | 25 mg kg$^{-1}$ MeXAA |
|---|---|---|
| Untreated | 23 ± 10 | 23 ± 10 |
| 2 h | 11 ± 2 | 15 ± 1 |
| 4 h | 6.1 ± 2* | 12 ± 1 |
| 24 h | Toxic | 6.3 ± 1.4* |

*$P<0.05$.

**Table V** The influence of 5,6-MeXAA on mice bearing intravenously administered MAC15A cells (median survival times)

| Treatment | Survival time (days) | Median survival time (days) | T C% |
|---|---|---|---|
| Control | 9, 10, 10, 10, 10 | 10 | |
| 5,6-MeXAA (30.0 mg kg$^{-1}$) | 0, 13, 16, 18, 18 | 17 | 170* |

*$P<0.01$.

It is clear that immunomodulation is involved in the mechanism of action of FAA in mice (reviewed by Bibby and Double, 1993), and the relevance of this to clinical disease remains to be established. Futami *et al.* (1991) compared the expression of cytokine genes in murine splenic leucocytes with human peripheral blood leucocytes, and their results demonstrated direct stimulation of cytokine gene expression in mouse but not in human leucocytes. They concluded that the failure of FAA to induce profound immunomodulation or therapeutic responses in man may relate to inherent differences in sensitivity between mouse and human cells. Preliminary data from this laboratory (Patel *et al.*, 1994) have demonstrated 5,6-MeXAA to be more effective than FAA at inducing TNF production from murine macrophages and splenocytes *in vitro*, and Ching *et al.* (1994) have demonstrated induction of TNF-α messenger RNA in human and murine cells, whereas FAA was effective only in murine cells. The relevance of these observations to possible *in vivo* activity in man needs to be addressed.

In conclusion, 5,6-MeXAA is substantially more potent than FAA *in vitro* against a mouse colon tumour cell line but is of only equal effectiveness against three human cell lines tested. The sensitive murine cell line produces established s.c. tumours which are highly responsive to 5,6-MeXAA in syngeneic hosts, but this activity is lost in nude hosts even though blood flow shutdown and haemorrhagic necrosis still occur. As with FAA there appears to be a very narrow therapeutic window, with activity being seen only close to maximum tolerated dose in each host. Anti-tumour effects are similar against s.c. tumours in NMRI mice. Maximum tolerated doses were marginally higher in NMRI than in nude hosts. Tumour cells inoculated i.p. fail to respond to the compound, even though *in vitro* studies showed the cells to be very sensitive to the compound. This is possibly due to insufficient drug exposure within the peritoneal cavity, although pharmacokinetic studies would be needed to substantiate this proposal. Clearly, as these cells grow as ascites, a vascular component cannot be involved. 5,6-MeXAA, unlike FAA, causes anti-tumour effects in an i.v. model, but the mechanism of action against this target appears different from that occurring against s.c. tumours and probably reflects the increased potency of the compound against this cell line. This increased potency will not be as useful against cell lines that are inherently resistant to 5,6-MeXAA, so a study of *in vitro* cytotoxicity against a number of cell lines reflecting different histological tumour types is being undertaken. A comparison of these data with *in vivo* activity against the same lines at clinically relevant anatomical sites might be useful for selecting likely clinical targets for this compound.

**Acknowledgements**
This work was supported by the Association for International Cancer Research and Bradford's War on Cancer.

**Table VI** The influence of 5,6-MeXAA on mice bearing intravenously administered MAC15A cells (colony counting method)

| Treatment | Number of colonies per mouse | Significance (Mann–Whitney) |
|---|---|---|
| Control | 43, 44, 53, 83, 94, 97, 100, 103 | |
| 5,6-MeXAA (27.5 mg kg$^{-1}$) | 21, 24, 27, 31, 32, 35, 55, 112, 137 | Not significant |
| 5,6-MeXAA (30.0 mg kg$^{-1}$) | 16, 20, 20, 21, 37, 40, 58, 86, 104 | $P<0.05$ |

**References**

ATWELL GJ, REWCASTLE GW, BAGULEY BC AND DENNY WA. (1989). Synthesis and antitumour activity of topologically related analogues of flavone acetic acid. *Anticancer Drug Design*, **4**, 161-169.

BIBBY MC AND DOUBLE JA. (1993). Flavone acetic acid - from laboratory to clinic and back. *Anticancer Drugs*, **4**, 3-17.

BIBBY MC, DOUBLE JA, PHILLIPS RM AND LOADMAN PM. (1987). Factors involved in the anticancer activity of the investigational agents LM985 (flavone acetic acid ester) and LM975 (flavone acetic acid). *Br. J. Cancer*, **55**, 159-163.

BIBBY MC, DOUBLE JA, PHILLIPS RM, LOADMAN PM AND GUMMER JA. (1988). Experimental anti-tumour effects of flavone acetic acid (LM975). Plant flavonoids in biology and medicine. II. Biochemical, cellular and medicinal properties. In *Progress in Clinical and Biological Research*, Vol. 280, Cody V, Middleton E, Harborne JB and Beretz A. (eds) pp. 243-246. Alan R Liss: New York.

BIBBY MC, PHILLIPS RM AND DOUBLE JA. (1989*a*). Influence of site on the chemosensitivity of transplantable murine colon tumours to flavone acetic acid (LM975, NSC 347512). *Cancer Chemother. Pharmacol.*, **24**, 87-94.

BIBBY MC, DOUBLE JA, LOADMAN PM AND DUKE CV. (1989*b*). Reduction of tumour blood flow by flavone acetic acid: a possible component of therapy. *J. Natl Cancer Inst.*, **81**, 216-220.

BIBBY MC, PHILLIPS RM, DOUBLE JA AND PRATESI G. (1991). Anti-tumour activity of flavone acetic acid (NSC 347512) in mice - influence of immune status. *Br. J. Cancer*, **63**, 57-62.

CAPOLONGO LS, BALCONI G, UBEZIO P, GIAVAZZI R, TARABOLETTI G, REGONESI A, YODER O AND D'INCALCI M. (1987). Antiproliferative properties of flavone acetic acid (NSC 347512) (LM975) a new anticancer agent. *Eur. J. Cancer Clin. Oncol.*, **23**, 1529-1535.

CARMICHAEL J, DEGRAFF WG, GAZDAR AF, MINNA JD AND MITCHELL JB. (1987). Evaluation of tetrazolium based semi-automated colorimetric assay: assessment of chemosensitivity testing. *Cancer Res.*, **47**, 936-942.

CHING L-M AND BAGULEY BC. (1987). Induction of natural killer cell activity by the antitumour compound flavone acetic acid (NSC 347512). *Eur. J. Cancer Clin. Oncol.*, **23**, 1047-1050.

CHING L-M, JOSEPH WR AND BAGULEY BC. (1992). Anti-tumour responses to flavone-8-acetic acid and 5,6-dimethylxanthenone-4-acetic acid in immune deficient mice. *Br. J. Cancer*, **66**, 128-130.

CHING L-M, JOSEPH WR, CROSIER KE AND BAGULEY BC. (1994). Induction of tumour necrosis factor-α messenger RNA in human and murine cells by the flavone acetic acid analogue 5,6 dimethylxanthenone-4-acetic acid (NSC 640488). *Cancer Res.*, **54**, 870-874.

CORBETT TH, BISSERY MC, WOZNIAK A, PLOWMAN J, POLIN L, TAPAZOGLOU E, DIEKMAN J AND VALERIOTE F. (1986). Activity of flavone acetic acid against solid tumours of mice. *Invest. New Drugs*, **4**, 207-220.

DOUBLE JA, BALL CR AND COWEN PN. (1975). Transplantation of adenocarcinoma of the colon in mice. *J. Natl Cancer Inst.*, **54**, 271-275.

EVELHOCH JL, BISSERY MC, CHABOT GG, SIMPSON NE, McCOY CL, HEILBRUN LK AND CORBETT TH. (1988). Flavone acetic acid (NSC 347512) induced modulation of murine tumour physiology monitored by *in vivo* nuclear magnetic resonance spectroscopy. *Cancer Res.*, **48**, 4749-4755.

FOGH J AND TREMPE G. (1975). New human tumour cell lines. In *Human Tumour Cells In Vitro*, Fogh J (ed.) pp. 119-154. Plenum: New York.

FUTAMI H, EADER LA, KOMSCHLIES KL, BULL R, GRUYS ME, ORTALDO JR, YOUNG HA AND WILTROUT RH. (1991). Flavone acetic acid directly induces expression of cytokine genes in mouse splenic leukocytes but not in human peripheral blood leukocytes. *Cancer Res.*, **51**, 6596-6602.

GERAN RI, GREENBERG NH, MacDONALD MM, SCHUMACHER AM AND ABBOT BJ. (1972). Protocols for screening chemical agents and natural products against tumour and other biological systems. *Cancer Chemother. Rep.*, **3**, 1-103.

HARADA M, TAKEUCHI M, FUKAO T AND KATAGIRI K. (1971). A simple method for the quantitative extraction of dye extravasated into the skin. *J. Pharm. Pharmacol.*, **23**, 218-219.

HILL S, WILLIAMS KB AND DENEKAMP J. (1989). Vascular collapse after flavone acetic acid: a possible mechanism of its anti-tumour action. *Eur. J. Cancer Clin. Oncol.*, **25**, 1419-1424.

HORNUNG RL, YOUNG HA, URBA WJ AND WILTROUT RH. (1988). Immunomodulation of natural killer cell activity by flavone acetic acid: occurrence via induction of interferon α/β. *J. Natl Cancer Inst.*, **80**, 1226-1231.

KERR DJ, MAUGHAN T, NEWLANDS E, RUSTIN G, BLEEHAN NM AND LEWIS C. (1989). Phase II trials of flavone acetic acid in advanced malignant melanoma and colorectal carcinoma. *Br. J. Cancer*, **60**, 104-106.

LOZZIO CB AND LOZZIO BB. (1975). Human chronic myelogenous leukaemia cell line with positive Philadelphia chromosome. *Blood*, **45**, 321-334.

McKEAGE MJ, KESTELL P, DENNY WA AND BAGULEY BC. (1991). Plasma pharmacokinetics of the anti-tumour agents 5,6-dimethylxanthenone-4-acetic acid, xanthenone-4-acetic acid and flavone-8-acetic acid in mice. *Cancer Chemother. Pharmacol.*, **28**, 409-413.

MAHADEVAN V, MALIK STA, MEAGER A, FIERS W, LEWIS GP AND HART IR. (1990). Role of tumour necrosis factor in flavone acetic acid induced tumour vascular shutdown. *Cancer Res.*, **50**, 5537-5542.

PATEL S, PARKIN SM AND BIBBY MC. (1994). The influence of flavone acetic acid and 5,6-dimethylxanthenone-4-acetic acid on the *in vitro* production of TNF from murine macrophages and splenocytes. *Br. J. Cancer*, **69**, 39.

PLOWMAN J, NARAYNAN VL, DYKES D, SZARVASI E, BRIET P, YODER OC AND PAULL KD. (1986). Flavone acetic acid: a novel agent with preclinical antitumour activity against colon adenocarcinoma 38 in mice. *Cancer Treat. Rep.*, **70**, 631-635.

PRATESI G, RODOLFO M, ROVETTA G AND PARMIANI G. (1990). Role of T cells and tumour necrosis factor in antitumour activity and toxicity of flavone acetic acid. *Eur. J. Cancer*, **26**, 1079-1083.

REWCASTLE GW, ATWELL GJ, BAGULEY BC, CALVELEY SB AND DENNY WA. (1989). Potential anti-tumour agents. 58. Synthesis and structure-activity relationships of substituted xanthenone-4-acetic acid against the colon 38 tumour *in vivo*. *J. Med. Chem.*, **32**, 793-799.

REWCASTLE GW, ATWELL GJ, ZHUANG L, BAGULEY BC AND DENNY WA. (1991*a*). Potential anti-tumour agents. 61. Structure-activity relationships for in vivo colon 38 activity among di-substituted 9-oxo-9*H*-xanthene-4-acetic acids. *J. Med. Chem.*, **34**, 217-222.

REWCASTLE GW, ATWELL GJ, PALMER BD, BOYD PDW, BAGULEY BC AND DENNY WA. (1991*b*). Potential antitumour agents. 62. Structure-activity relationships for tricyclic compounds related to the colon tumour active drug 9-oxo-9*H*-xanthene-4-acetic acid. *J. Med. Chem.*, **34**, 491-496.

REWCASTLE GW, ATWELL GJ, BAGULEY BC, BOYD M, THOMSEN LL, ZHUANG L AND DENNY WA. (1991*c*). Potential antitumour agents. 63. Structure-activity relationships for side-chain analogues of the colon 38 active agent 9-oxo-9*H*-xanthene-4-acetic acid. *J. Med. Chem.*, **34**, 2864-2870.

SCHROYENS WA, DODION PP, SANDERS C, LOOS M, DETHIER NE, DELFORGE AR, STRYCHMANS PA AND KENIS Y. (1987). *In vitro* chemosensitivity testing of flavone acetic acid (LM975 NSC347512) and its diethylaminoethyl ester derivative (LM985 NSC293015). *Eur. J. Cancer Clin. Oncol.*, **23**, 1135-1139.

THOMSEN LL, CHING L-M AND BAGULEY BC. (1990). Evidence for the production of nitric oxide by activated macrophages treated with the anti-tumour agents flavone-8-acetic acid and xanthenone-4-acetic acid. *Cancer Res.*, **50**, 6966-6970.

THOMSEN LL, CHING LM, ZHUANG L, GAVIN JB AND BAGULEY BC. (1991). Tumour dependent increased plasma nitrate concentrations as an indication of the antitumour effect of flavone-8-acetic acid and analogues in mice. *Cancer Res.*, **51**, 77-81.

THOMSEN LL, BAGULEY BC, CHING L-M AND GAVIN JB. (1992). Modulation of superoxide production from murine macrophages by the anti-tumour agent flavone acetic acid and xanthenone acetic acid analogues. *Biochem. Pharmacol.*, **43**, 386-389.

TOMPKINS WAF, WATRACH AM, SCHMALE JB, SCHULTZ RM AND HARRIS JA. (1974). Cultural and antigenic properties of newly established cell strains derived from adenocarcinomas of the human colon and rectum. *J. Natl Cancer Inst.*, **52**, 1101-1111.

VESZELOVSZKY E, THOMSEN LL, ZHUANG L AND BAGULEY BC. (1993). Flavone acetic acid and 5,6-dimethylxanthenone-4-acetic acid: relationship between plasma nitrate elevation and the induction of tumour necrosis. *Eur. J. Cancer*, **29**, 404-408.

WILTROUT RH, BOYD MR, BACK TC, SALUP RR, ARTHUR JA AND HORNUNG RL. (1988). Flavone-8-acetic acid augments systemic natural killer cell activity and synergises with IL-2 for treatment of murine renal cancer. *J. Immunol.*, **140**, 3261-3265.

ZWI JL, BAGULEY BC, GAVIN JB AND WILSON WR. (1989). Blood flow failure as a major determinant in the anti-tumour action of flavone acetic acid. *J. Natl Cancer Inst.*, **81**, 1005-1013.